# Sociodemographic and health risk factors associated with health-related quality of life among adults living in Puerto Rico in 2019: a cross-sectional study

**DOI:** 10.1186/s12889-023-17115-3

**Published:** 2023-11-03

**Authors:** Irene Frontera-Escudero, José A. Bartolomei, Alejandro Rodríguez-Putnam, Luz Claudio

**Affiliations:** 1 Department of Environmental Medicine and Public Health, Division of International Health, Icahn School of Medicine at Mount Sinai, New York, NY USA; 2 Outcome Project, LLC, Vega Baja, Puerto Rico; 3 Department of Health Services Administration, Graduate School of Public Health, University of Puerto Rico Medical Sciences Campus, San Juan, Puerto Rico

**Keywords:** HRQoL, Puerto Rico, Health disparities, Population health, Chronic Disease, BRFSS, Public health, Social determinants of health

## Abstract

**Background:**

Puerto Rico, a US territory, faces numerous challenges adversely affecting public health, including poverty, a fragile healthcare system, inadequate infrastructure, a debt crisis, and vulnerability to climate change-related natural disasters. The impact of these factors on the Health-Related Quality of Life (HRQoL) measure has not been comprehensively evaluated. Only two studies have assessed HRQoL, with the latest conducted in 2011, prior to recent events that could affect public health. This study aimed to assess the HRQoL and associated sociodemographic and health risk factors among adults living in Puerto Rico in 2019.

**Methods:**

Prevalence and 95% confidence intervals were used to describe HRQoL and its associations with sociodemographic and health-related variables among adults living in Puerto Rico who answered the Behavioral Risk Factor Surveillance System (BRFSS) survey (n = 4,944) in 2019. Multivariable logistic regression models were developed to identify which of these variables were more likely to be associated with each of the four core HRQoL questions (HRQoL-4), expressed as prevalence odds ratios with 95% confidence intervals adjusted for potential confounders.

**Results:**

Through a comprehensive multivariable analysis, we uncovered significant risk factors – increasing number of chronic conditions, advanced age, and low income – associated with *poor HRQoL* among adults living in Puerto Rico. Specifically, our findings suggest that individuals with an increasing number of chronic conditions were more likely to report *poor HRQoL* across all 4 domains. As the number of reported chronic conditions increases by one, the odds of reporting having: fair/poor general health increased by a factor of 2.24 (POR: 2.24, 95% CI: 2.08–2.41), physical health impairment increased by a factor of 1.93 (POR: 1.93, 95% CI: 1.78–2.08), mental health impairment increased by a factor of 1.90 (POR: 1.90, 95% CI: 1.78–2.02) and activity limitation increased by a factor of 1.27 ( POR: 1.27, 95% CI: 1.13–1.42). Advancing age was associated with all domains of *poor HRQoL*, except for the mental health domain for which we observed higher rates of *poor HRQoL* among the younger population (POR: 4.76, 95% CI: 2.4–9.1).

**Conclusion:**

This paper shows that the prevalence of *poor HRQoL* has not improved compared to the only previous study of HRQoL of Puerto Rico in the last decade. We also found that *poor HRQoL* is associated with having multiple chronic conditions in adults living in Puerto Rico. This may be a consequence of a decline in health services after natural disasters and socioeconomic downturns on the island. The study emphasizes the need for targeted interventions and ongoing monitoring of the population’s HRQoL over time to reach vulnerable subgroups, especially those with chronic conditions, advanced age, and low income, in order to reduce health disparities in Puerto Rico.

## Background

The residents of Puerto Rico (a US territory) are confronted with a multitude of challenges that affect public health, including high levels of poverty, a fragile healthcare system, inadequate infrastructure, a debt crisis, and climate change vulnerability [1, 2]. Public health interventions, such as improved access to physical and mental healthcare and chronic disease management designed to reach vulnerable subgroups might help adults in Puerto Rico increase their quality and years of healthy life and reduce health disparities [3]. Puerto Rico faces income and wealth inequality, unemployment, outmigration, and limited access to basic health and social welfare infrastructure. These challenges have worsened recently due to fiscal mismanagement, debt crisis, hurricanes Irma and Maria in 2017, and multiple earthquakes in 2019 and 2020. [4]. According to the Census Bureau [5], poverty affects 43.5% of Puerto Ricans, with a median household income of $20,539 compared to $67,521 in the US. These inequities exacerbate adverse health outcomes affecting Puerto Ricans’ well-being and Health-Related Quality of Life (HRQoL) at population levels [4].

Demographic, socioeconomic, behavioral, and socio-environmental factors affect HRQoL, a multidimensional concept that the Centers for Disease Control and Prevention (CDC) defined as “an individual’s or group’s perceived physical and mental health over time” [6]. To measure HRQoL, the CDC developed and validated a set of four core questions that they called “Healthy Days Measures” (HRQoL-4). The HRQoL-4 questions assess four domains: self-reported general health, physical health, mental health, and activity limitations. Studying HRQoL, an encompassing measure of health perception, offers valuable insights into overall health status, health disparities, and healthcare requirements at the population level [7], enabling a comprehensive understanding of health-related issues, the equitable distribution of care, and the broader health landscape [6].

Several recent federal policy changes underscore the need to measure HRQoL to supplement public health’s traditional measures of morbidity and mortality because these measures fall short of incorporating the voice of individuals [6]. By incorporating the voice of individuals, the CDC recognizes the importance of gathering data directly from people themselves to understand their subjective experiences and how health conditions affect their overall quality of life. In addition, HRQoL has been used to determine the burden of preventable diseases and disabilities in the population and can provide valuable new insights into the relationships between HRQOL and risk factors. [6]. Its importance has led to a growing consensus for evaluating HRQoL in public health surveillance systems and intervention studies [6]. *Healthy People 2000, 2010, and 2020* identified quality of life improvement as a central public health goal [6, 9]. Also, tracking population HRQoL over time helps identify health disparities, evaluate progress on achieving broad health goals, and inform healthy public policy [10]. Focusing on HRQoL as an outcome can bridge boundaries between disciplines and social, mental, and medical services because this measure incorporates personal subjective experiences that can affect health conditions and overall perception of health in populations [6, 11].

There have only been two studies of HRQoL in adults living in Puerto Rico that utilize the Behavioral Risk Factors Surveillance System (BRFSS) [3, 12]. The last assessment was conducted in 2011 [12], before many events, such as natural disasters and economic strife, which may have caused changes in this index. The objective of this study is to assess determined HRQoL of adults residing in Puerto Rico in 2019 and the association between identified risk factors and social determinants of health with HRQoL.

## Methods

The study design involved conducting a retrospective analysis of cross-sectional data obtained from the 2019 BRFSS. The main objective was to assess the factors associated with HRQoL.

### Participants

Our study sample included 4,944 individuals 18 + years of age living in Puerto Rico who responded to the BRFSS survey in 2019. This study used publicly available data, and institutional review board approval was not required.

### Data source and study sample

Data for this study were obtained from the 2020 Behavioral Risk Factor Surveillance System (BRFSS) [13]. The BRFSS is an annual cross-sectional, ongoing, state-based, random digit–dialed health telephone survey for non-institutionalized adults aged 18 years or older residing in the United States [14]. This survey collects information from a representative sample of adults in each US state and its territories about health risk behaviors, clinical preventive health practices, and health care access, primarily related to chronic disease and injury [14]. The BRFSS also includes the CDC’s “Healthy Days Measure” (HRQoL-4), a set of questions about perceived HRQoL [6]. Further information about survey design and sampling method can be found on the BRFSS website (http://www.cdc.gov/BRFSS) [14]. Our study sample included all 4,944 individuals 18 + years of age living in Puerto Rico who responded to the BRFSS survey in 2019.

### Materials

Data for this study were obtained from the 2020 Behavioral Risk Factor Surveillance System (BRFSS) [13]. The BRFSS is an annual cross-sectional, ongoing, state-based, random digit–dialed health telephone survey for non-institutionalized adults aged 18 years or older residing in the United States [14]. This survey collects information from a representative sample of adults in each US state and its territories about health risk behaviors, clinical preventive health practices, and health care access, primarily related to chronic disease and injury [14]. The BRFSS also includes the CDC’s “Healthy Days Measure” (HRQoL-4), a set of questions about perceived HRQoL [6]. Further information about survey design and sampling method can be found on the BRFSS website (http://www.cdc.gov/BRFSS) [14].

### Dependent variable

#### Health-related quality of life (HRQoL-4)

HRQoL was assessed by the following four core questions called “Healthy Days Measures” (HRQoL-4) developed by the CDC [6]: (1) General Health: “Would you say that in general your health is: excellent, very good, good, fair, or poor?”; (2) Physical Health: “Now thinking about your physical health, which includes physical illness and injury, for how many days during the past 30 days was your physical health not good?”; (3) Mental Health: “Now thinking about your mental health, which includes mental illness, for how many days during the past 30 days was your mental health not good?”; and (4) Activity limitation: “During the past 30 days, for about how many days did poor physical or mental health keep you from doing your usual activities, such as self-care, work, or recreation?”. General health was further categorized as fair/poor versus excellent/ very good/good. The other three HRQoL measures (physical health, mental health, and activity limitation) were dichotomized into 14 days or more versus 0 to 13 days, as previously used in other studies [8, 15]. *Poor HRQoL* was defined as fair/poor for general health or 14 days or more for each of the other three HRQoL measures.

### Independent variables

#### Sociodemographic characteristics and chronic conditions

Sociodemographic variables included in this study were: age (18–34, 35–64, 65 < years), gender (female/male), education level (never attended school, elementary, some high school, high school graduate, some college or technical school, college graduate, refused), employment status (employed for wages, self-employed, out of work for one year or more, out of work for less than one year, homemaker, student, retired, unable to work, refused), income (less than $10,000, less than $15,000, less than $20,000, less than $25,000, less than $35,000, less than $50,000, less than $75,000, $75,000 or more, don’t know/not sure, refused). Another variable was created to measure the number of self-reported chronic conditions a respondent has using the following health-related variables: heart attack, angina or coronary heart disease, stroke, skin cancer, any other types of cancer, chronic obstructive pulmonary disease, C.O.P.D., emphysema or chronic bronchitis, arthritis, rheumatoid arthritis, gout, lupus, or fibromyalgia, depressive disorder (including depression, major depression, dysthymia, or minor depression), kidney disease (not including kidney stones, bladder infection or incontinence), current asthma (where adults response options for this chronic condition were yes, no, don’t know/ not sure, refused) and diabetes (where adults response options for this chronic condition were yes, yes, but female told only during pregnancy, no, no, pre-diabetes or borderline diabetes, don’t know/not sure, refused).

### Procedures

The 2020 BRFSS data was obtained by directly downloading it from the official CDC website. The statistical package R was then utilized to select the specific variables.

### Statistical analysis

Frequencies, weighted frequencies, proportions and 95% confidence intervals (CI) were used to describe the sample characteristics and population distributions [9]. To describe HRQoL among the population under study, we assessed bivariate analysis between sociodemographic and health-related variables with HRQoL-4 domains using weighted prevalence estimates and 95% CI. Multivariable logistic regression model methodologies [8, 16] were fitted to identify which variables were associated with each of the HRQoL-4 domains. The model tested for interaction and adjusted for confounding variables (such as age, sex, household income, and chronic conditions). The quantification of missing data was carried out, ensuring that it did not significantly impact or distort the results. The final model for each HRQoL-4 domain was expressed as the estimated adjusted prevalence odds ratios (POR) with 95% CI. POR and 95% CI less than 1 are interpreted as their inverse (1/x**)** [17]. For each of the sociodemographic variables included in the multivariable logistic regression model of the study, a distinct category served as a reference group: in age, it was adults aged 18–34; in gender, it was male; and in household income, it was $75,000 or more. We tested education and employment variables in our model; however, they were not included in our final analysis. We did not observe any significant difference in the PORs, and none of the education and employment status categories increased the likelihood of *poor HRQoL* compared to the reference group. All analyses were conducted in R version 4.0 using the *complex sampling survey* package (v4.0) [18] to estimate the 95% CI, incorporating the BRFSS sampling design [14].

## Results

Descriptive characteristics of the study population (4,944) are in Table 1, showing their representativeness of the overall adult population in Puerto Rico (2,717,794) [19], which coincides with BRFSS data weighted estimates (N = 2,737,636). The Census Bureau also reported that 52.5% of the population in Puerto Rico were female [19]. In BRFSS, population estimates of females were 53.3%. Comparison between both questionnaires validates that the BRFSS collected an unbiased sample regarding estimated population distribution in terms of sex and age in Puerto Rico during the years 2019 and 2020.

**Table 1:** Demographic Characteristics of Adults Living in Puerto Rico in 2019, BRFSS

Characteristics	Overall Sample: Adults living in Puerto Rico				
	Unweighted n = 4,944	Weighted N = 2,737,636			
	Frequency	Population Estimate	Proportion	95% C.I.	
Sex	n*=4,944				
Male	1978	1,278,659	46.7	44.5–49	
Female	2966	1,458,977	53.3	51–55.5	
Age	n*=4,944				
18–24	390	346,845	12.7	11.3–14.1	
25–34	747	434,128	15.9	14.4–17.3	
35–44	785	438,751	16	14.5–17.5	
45–54	955	455,380	16.6	15.3–18	
55–64	956	432,911	15.8	14.4–17.2	
65+	1111	629,621	23	20.8–25.2	
Employment Status	n*=4,903				
Employ for Wages	1701	864,186	31.78	30–33.56	
Self-employed	628	340,603	12.53	11.33–13.72	
Out of work > 1 year	232	153,888	5.66	4.41–6.91	
Out of work < than 1 Year	265	151,915	5.59	4.71–6.46	
A homemaker	666	384,190	14.13	12.48–15.77	
A student	163	124,113	4.56	3.75–5.38	
Retired	975	534,592	19.66	17.51–21.81	
Unable to work	273	165,975	6.1	4.92–7.28	
Education	n*=4,934				
Never attended	17	17,883	0.65	0.29–1.02	
Elementary	302	328,292	12.01	9.89–14.14	
Some high school	263	295,829	10.82	9.07–12.58	
High school graduate	1189	756,977	27.69	25.77–29.62	
Some college	1248	452,959	16.57	15.36–17.78	
College graduate	1915	881,507	32.25	30.50–34	
Household Income	n*=3,973				
<$10,000	851	607,761	27.76	25.21–30.3	
$10,000 - <$15,000	576	333,752	15.24	13.39–17.1	
$15,000 - <$20,000	675	349,439	15.96	14.44–17.48	
$20,000 - <$25,000	601	302,395	13.81	11.98–15.64	
$25,000 - <$35,000	447	204,712	9.35	8.19–10.51	
$35,000 - <$50,000	325	160,733	7.34	6.39–8.3	
$50,000 - <$75,000	242	108,231	4.94	4.21–5.68	
$75,000 or more	256	122,525	5.6	4.77–6.42	
Chronic conditions	n*=4,944				
0 conditions	2482	1,404,797	51.31	49.2–53.4	
1 condition	1257	671,627	24.53	22.7–26.4	
2 conditions	665	354,371	12.94	11.4–14.5	
3 conditions	304	179,687	6.56	4.98–8.1	
4 conditions	128	65,338	2.38	1.8-3.0	
5 conditions	56	35,763	1.3	0.6-2.0	
6 conditions	36	18,652	0.68	0.4–0.96	
7 conditions	10	4,537	0.16	0.04–0.3	
8 conditions	6	2,864	0.1	0.01–0.2	
**HRQoL measures**					
General Health	n*=4,932				
Excellent/VeryGood/Good	3686	1,998,832	73	71–75	
Fair/Poor	1246	732,824	27	25–29	
Physical Health	n*=4,897				
0–13 days	4339	2,386,388	88	86–90	
14 + days	558	326,753	12	10–14	
Mental Health	n*=4,877				
0–13 days	4303	2,391,001	89	87.8–90	
14 + days	574	296,720	11	9.8–12	
Activity Limitation	n*=1,670				
0–13 days	1352	747,679	81	78–84	
14 + days	318	177,346	19	16–22	

*Notes*: n*= unweighted frequency total of respondents that answered that specific question

Table 1 also indicates that the groups with the highest proportions were adults 65 years or older (23%), married (36.92%), and graduated from college (32%). Additionally, the respondents’ most prevalent employment status and annual household income were employed for wages (31.78%) and annual household income below $10,000 (27.76%). Regarding the number of chronic conditions, they ranged from 0 to 8, with a median of one chronic condition and with 49% reporting having at least one chronic condition. The percentage of respondents categorizing their HRQoL as *poor* in the different domains of the metric was 27% in the general health domain, 12% in physical health, 11% in mental health, and 19% in activity limitation.

Table 1. Demographic Characteristics of Adults Living in Puerto Rico in 2019, BRFSS *(See attached* Table 1* at page 27–28).*

Bivariate relationships between the sociodemographic variables and each of the four domains of HRQoL show that across all the HRQoL-4 domains, the majority of the population, in terms of sex and age, were significantly more likely to report having excellent/very good/ good in general health and less than 14 days of physical health impairment, mental health impairment and activity limitation (Table 2). Nevertheless, in the older populations, all HRQoL-4 domains presented an increase in the prevalence of *poor HRQoL*. Individuals who reported being out of work, homemakers, retired or unable to work were more likely to report having *poor HRQoL* as compared to individuals who reported being a student or employed. Additionally, as individuals reported lower household income and/or lower education, they were more likely to report having *poor HRQoL.*

**Table 2:** Prevalence and 95% Confidence Intervals of HRQoL-4 Domains by Sociodemographic Characteristics

Characteristics	General Health		Physical Health		Mental Health		Activity Limitation	
	Excellent/Very good/ Good N*=1,998,832 P* (95% CI)	Fair/ Poor N*=732,824 P* (95% CI)	0–13 days N*=2,386,388 P* (95% CI)	14 + days N*= 326,753 P* (95% CI)	0–13 days N*= 2,391,001 P* (95% CI)	14 + days N*= 296,720 P* (95% CI)	0–13 days N*= 747,679 P* (95% CI)	14 + days N*= 177,346 P* (95% CI)
Sex								
Male	77 (74–80)	23 (20–26)	90 (87–92)	10 (7.9–13)	91 (89–93)	9 (7.2–11)	77 (71–84)	23 (16–29)
Female	70 (67–73)	30 (27–33)	86 (84–89)	14 (10.8–16)	87 (86–89)	13 (11.3–14)	83 (80–86)	17 (14–20)
Age								
18–24	93 (89–96)	7.5 (4.4–10.5)	98 (97–99)	2.1 (0.7–3.5)	92 (89–95)	7.8 (4.9–10.8)	92 (86–97)	8.2 (2.9–14)
25–34	93 (90–96)	7 (4.1–9.8)	95 (93–98)	4.7 (2.2–7.2)	90 (86–93)	10.4 (7.1–13.6)	88 (81–95)	12.2 (5.3–19
35–44	86 (82–89)	14.4 (10.9–17.9)	91 (85–96)	9.4 (4.3–14.5)	87 (83–89)	12.6 (9.6–15.6)	87 (82–92)	13.1 (8–18)
45–54	72 (69–76)	27.6 (23.8–31.4)	88 (86–91)	11.7 (8.9–14.4)	86 (83–89)	14.2 (11.3–17.1)	78 (72–83)	22.2 (16.9–28)
55–64	60 (56–64)	40 (35.6–44.5)	81 (78–84)	19.1 (15.7–22.4)	85 (82–88)	15.2 (12.3–18.1)	73 (67–79)	26.9 (21.2–33)
65+	50 (43–56)	50.3 (43.9–56.7	80 (74–86)	20.1 (14.1–26.1)	93 (91–95)	7 (4.6–9.3)	77 (67–87)	23.2 (13–33)
Employment Status								
Employ for Wages	90 (88–92)	9.9 (8.2–11.7)	94 (93–96)	5.7 (4.3–7.1)	92 (90–94)	8.1 (6.5–9.6)	90 (87–93)	10.1 (6.99-13)
Self-employed	86 (82–89)	14.3 (10.6–18)	95 (93–97)	5.4 (3.38–7.4)	90 (87–93)	10.2 (7-13.4)	83 (76–90)	17.2 (10.42-24)
Out of work > 1 year	63 (51–76)	36.5 (24.2–48.8)	80 (67–92)	20.3 (7.56–33.1)	91 (87–94)	9.5 (5.5–13.4)	58 (34–82)	41.6 (17.76-66)
Out of work < than 1 Year	85 (79–91)	15.4 (9.4–21.5)	92 (88–96)	8.1 (4.25-12)	86 (79–92)	14.3 (7.6–21)	80 (69–91)	19.9 (8.7–31)
A homemaker	62 (57–68)	37.8 (32.3–43.2)	84 (78–90)	16 (10.09-22)	89 (86–92)	11.2 (8.2–14.1)	87 (82–92)	13 (7.82-18)
A student	94 (90–98)	6 (2.3–9.7)	98 (96–100)	1.7 (-0.25-3.7)	91 (87–96)	8.6 (4.1–13.1)	90 (82–99)	9.6 (0.83-18)
Retired	51 (44–57)	49.4 (42.8–55.9)	81 (75–87)	19.1 (12.99–25.1)	90 (87–93)	10 (6.9–13.2)	79 (72–87)	20.7 (13.08-28)
Unable to work	40 (29–51)	60.1 (49.2–71.1)	69 (60–77)	31.4 (22.96–39.8)	68 (59–76)	32.3 (23.8–40.7)	63 (53–72)	37.5 (27.87-47)
Education								
Never attended	28 (2.4–54)	72 (46–98)	70 (45–96)	29.7 (4-55.4)	74 (46–102)	26 (-1.8-54)	72 (42–103)	28 (-2.5-58)
Elementary	45 (35.6–55)	55 (45–64)	74 (63–84)	26 (15.5–36.5)	92 (88–95)	8.4 (4.7–12)	78 (62–94)	22 (6.3–38)
Some high school	56 (46.2–66)	44 (34–54)	87 (83–91)	13 (8.5–17.5)	93 (89–96)	7.2 (3.9–11)	81 (72–89)	19 (10.7–28)
High school graduate	71 (68–75)	29 (25–32)	87 (83–90)	13.1 (9.5–16.6)	87 (85–90)	12.5 (9.9–15)	78 (72–83)	22 (16.8–28)
Some college	82 (79.8–85)	18 (15–20)	90 (88–92)	9.8 (7.9–11.7)	89 (86–91)	11.3 (9–14)	81 (76–86)	19 (14.5–24)
College graduate	87 (85.4–89)	13 (11–15)	93 (92–95)	6.5 (5.3–7.8)	88 (87–90)	11.6 (9.8–13)	86 (83–89)	14 (10.6–17)
Household Income								
<$10,000	58 (52–65)	41.6 (35.5–47.7)	82 (77–87)	18 (12.9–23.1)	87 (84–90)	13.1 (10.2–16)	75 (66–84)	25.1 (16.2–34)
$10,000 - <$15,000	67 (61–73)	32.9 (27.3–38.6)	85 (81–89)	15 (10.78–19.1)	82 (77–86)	18.2 (13.7–22.8)	78 (71–85)	21.8 (14.6–29)
$15,000 - <$20,000	76 (72–80)	24 (20.1–27.9)	88 (85–91)	11.7 (8.85–14.6)	91 (88–93)	9.4 (6.9–12)	78 (72–85)	21.7 (15.2–28)
$20,000 - <$25,000	76 (67–85)	24.1 (15.1–33.1)	88 (78–97)	12.3 (2.6–22.1)	92 (89–94)	8.2 (5.7–10.7)	87 (81–94)	12.6 (6.4–19)
$25,000 - <$35,000	86 (82–90)	14 (9.6–18.3)	94 (91–96)	6.4 (3.67–9.2)	89 (84–93)	11.1 (6.7–15.6)	86 (79–93)	14.4 (7.4–21)
$35,000 -<$50,000	91 (88–95)	8.6 (5.3–11.9)	95 (92–98)	5 (2.41–7.5)	90 (85–94)	10.2 (5.5–14.8)	92 (86–98)	7.7 (1.8–14)
$50,000 - <$75,000	92 (89–96)	7.9 (4.4–11.3)	96 (93–98)	4.4 (1.87–6.9)	95 (92–97)	5.5 (2.5–8.4)	88 (79–97)	11.8 (2.8–21)
$75,000 or more	97 (95–99)	3.2 (1.3–5.1)	96 (92–99)	4.5 (0.77–8.2)	95 (92–97)	5.4 (2.6–8.2)	93 (86–99)	7.1 (0.6–14)

*Notes*:

N*= total weighted frequency of respondents within specific HRQoL domain

P*= Prevalence

95% CI = Confidence Interval

Table 2. Prevalence and 95% Confidence Intervals of HRQoL-4 Domains by Sociodemographic Characteristics (*See attached* Table 2* at page 29–31).*

Table 3 displays the adjusted multivariable logistic regression estimates of sociodemographic variables and chronic conditions with each of the four domains of HRQoL(general health, physical health impairment, mental health impairment, activity limitation) .

### General Health

In the older age groups, adults living in Puerto Rico were more likely to report having poor general health. The highest difference observed between age groups was in people aged 65 + who were 6.24 times more likely to report fair/poor general health than people aged 18–24 (POR: 6.24, 95% CI: 3.17–12.3). Lower income adults living in Puerto Rico were more likely to report having fair/poor general health. Adults with an income of <$10,000 had 14.4 times higher odds of reporting fair/poor general health compared to adults with an income of $75,000 or more (POR: 14.4, 95% CI: 7.02–29.89). We found that in this population, as the number of reported chronic conditions increases by one, the odds of reporting having fair/poor general health increased by a factor of 2.24 (POR: 2.24, 95% CI: 2.08–2.41).

### Physical Health Impairment

The odds of reporting physical health impairment ranged between 2 and 3 times higher in older age groups when compared to people aged 18 to 24. Lower income groups were more likely to report having physical health impairment. Adults with an income of <$10,000 had 2.58 times the odds of reporting physical health impairment compared to people with an income of $75,000 or more (POR: 2.58, 95% CI: 1.62–3.55). As the number of reported chronic conditions increases by one, the odds of reporting having physical health impairment increased by a factor of 1.93 (POR: 1.93, 95% CI: 1.78–2.08).

### Mental Health Impairment

The highest difference observed between age groups was in people aged 18 to 24, which were 4.76 times more likely to report mental health impairment than people aged 65+ (POR: 4.76, 95% CI: 2.4–9.1). People with an income group of <$50,000 were more likely to report mental health impairment than the reference group. The largest difference observed between income groups was in people with an income of $10,000 to <$15,000, who had 3 times higher odds of reporting mental health impairment compared to people with an income of $75,000 or more (POR: 3.40, 95% CI: 2.74–4.06). With increasing number of reported chronic conditions, the odds of reporting having mental health impairment increased by a factor of 1.90 for each chronic condition (POR: 1.90, 95% CI: 1.78–2.02).

### Activity limitation

Adults aged 55 to 64 had 3.12 higher odds of reporting activity limitation than adults aged 18 to 24. Meanwhile, people with an income of <$10,000, $10,000- <$15,000 and $15,000 -<$25,000 were 2 to 3 times more likely to report having activity limitations than people with an income of $75,000 or more (POR: 3.24, 95% CI: 2.13–4.35; POR: 2.79, 95% CI: 1.68–3.91; POR: 3.26, 95% CI: 2.17–4.36). Lastly, as the number of reported chronic conditions increased by one, the odds of reporting having activity limitation increased by a factor of 1.27 (POR: 1.27, 95% CI: 1.13–1.42).

**Table 3:** Adjusted Prevalence Odds Ratios (and 95% Confidence Intervals) from Multivariable Logistic Regression for HRQoL-4 Domains by Sociodemographic Characteristics and Chronic Conditions

Characteristics	Fair/Poor General Health	Physical Health Impairment 14 + days	Mental Health Impairment 14 + days	Activity Limitation 14 + days	
Sex					
Male (Ref)	Ref	Ref	Ref	Ref	
Female	1.03 (0.78–1.38) *	0.98 (0.64–1.52)	1.37 (1.02–1.86) *	0.65 (0.4–1.07)	
Age					
18–24 (Ref)	Ref	Ref	Ref	Ref	
25–34	1.44 (0.68–3.06)	2.19 (0.84–5.75)	1.34 (0.72–2.51)	1.79 (0.62–5.17)	
35–44	2.2 (1-4.84) *	3.74 (1.52–9.2) *	1.44 (0.76–2.73) *	1.57 (0.59–4.2)	
45–54	4.47 (2.33–8.61) *	3.34 (1.45–7.74) *	1.24 (0.68–2.25) *	2.71 (1.08–6.81) *	
55–64	5.16 (2.68–9.96) *	3.53 (1.54–8.14) *	0.87 (0.47–1.61) *	3.12 (1.24–7.87) *	
65+	6.24 (3.17–12.3) *	2.93 (1.16–7.41) *	0.21 (0.11–0.42)	1.75 (0.56–5.52) *	
Household Income					
<$10,000	14.4 (7.02–29.89) *	2.58 (0.98–6.79) *	1.90 (0.99–3.68) *	3.24 (1.07–9.82) *	
$10,000 - <$15,000	10.3 (5.02–21.28) *	2.36 (0.9–6.2) *	3.40 (1.76–6.57) *	2.79 (0.92–8.49) *	
$15,000 - <$20,000	8.59 (4.25–17.35) *	2.22 (0.88–5.67) *	1.57 (0.81–3.06)	3.26 (1.09–9.75) *	
$20,000 - <$25,000	6.52 (3.03–14.05) *	2.00 (0.64–6.34)	1.26 (0.64–2.51)	1.54 (0.47–5.09)	
$25,000 - <$35,000	3.80 (1.74–8.32) *	1.05 (0.38–2.95)	1.80 (0.88–3.69) *	1.95 (0.62–6.21)	
$35,000 - <$50,000	2.77 (1.24–6.24) *	0.96 (0.34–2.73)	1.74 (0.82–3.74)	0.96 (0.26–3.53)	
$50,000 - <$75,000	2.42 (1.08–5.43) *	0.87 (0.3–2.53)	0.91 (0.41–2.05)	1.54 (0.4–5.97)	
$75,000 or more (Ref)	Ref	Ref	Ref	Ref	
Chronic Conditions					
Inreases by one	2.24 (1.9–2.65) *	1.93 (1.66–2.25) *	1.90 (1.69–2.15) *	1.27 (1.11–1.47) *	

*Notes*: POR = Prevalence Odds Ratio, CI = 95% Confidence Interval, Ref = Reference group, * = p < 0.05

POR and 95% CI values less than 1 are interpreted as their inverse (1/x) [17]

## Discussion

The results of this study suggest that sociodemographic characteristics and chronic health conditions are associated with specific measures of HRQoL among adults living in Puerto Rico. Based on the regression models, we identified that individuals with increasing age and low income are at a higher risk of reporting *poor HRQoL*. The number of increasing chronic conditions are also associated with *poor HRQoL* reducing the magnitude of association of age and income with *poor HRQoL*, even after accounting for confounding factors. This was consistent except for younger adults, who are more likely to report having *poor HRQoL* in the mental health domain. In addition, our study results on the prevalence of people rating their HRQoL as *poor* show that it has not improved compared to the only previous study of HRQoL of Puerto Rico in the last decade [12], specifically, the prevalence of people rating their HRQoL as *poor* increased in the activity limitation domain and has not improved in the physical and mental health domains.

In general, a high prevalence of chronic disease persisted for Puerto Ricans on the island as well as on the US mainland [20, 21]. However, comparisons between Puerto Ricans on the island and on the US mainland show marked differences in factors such as higher health care coverage and educational attainment but lower income on the island [22]. Consistent with other studies [22–24], our results show that the odds of reporting *poor HRQoL* increased significantly and consistently in all the four domains with an increasing number of self-reported chronic conditions and that this association persisted following adjustment for confounding factors. Our results show that considering chronic conditions and adjusting for this variable decreased the association (odds ratio) between age, income, and poor *HRQoL*. This implies that chronic conditions have a substantial impact on HRQoL and play a confounding role, suggesting that chronic conditions are a strong predictor of *poor HRQoL*. *Poor HRQoL* is known to be a significant consequence of multimorbidity; in particular, this is because people who have several conditions might need to visit many providers and follow complex treatment recommendations [24]. Approximately a third of adults worldwide suffer from multiple chronic conditions, resulting in high healthcare expenses and increased prescription drug use for each additional chronic condition [25]. This burden contributes to a decline in quality of life for patients, financial strain, medication adherence issues, inability to work, and symptom management challenges [25].

Compared with other US Hispanics, Puerto Ricans overall have higher education levels [5]; however, Puerto Ricans have lower median household incomes, lower homeownership rates, and are more likely to be poor [26]. Almost 50% of Puerto Rico’s population lives in poverty as the median household income is $20,539, compared to $67,521 in the US [5]. The compounding effects of island-wide poverty and a high prevalence of underlying risk factors among older adults threaten an already unstable economic structure reeling from funding shortfalls and lack of healthcare access [4, 27].

In our study, as in other previous studies, age is an important factor associated with *poor HRQoL*. Specifically, age 45–65 + was a predictor of *poor HRQOL*, with levels increasing as individuals age [28, 29], particularly in the general and physical health impairment domains. A reduction in HRQoL as age increases highlights a missed opportunity for early medical intervention and the need for continued treatment [30]. The relationship between increasing age and *poor HRQoL* was consistent with previous studies, except for the mental health domain, for which individuals aged 65 years or older had the lowest rates. This finding is consistent with previous studies that showed that older adults appear to have the lowest rate of mentally unhealthy days as compared to younger adults [8, 31]. In addition, comparing our study’s results to the previous HRQoL study conducted in Puerto Rico in 2011 [12 ], our study revealed that the young adult population (18–35) had the highest rates of mental health impairment. This increase in rates of mental health impairment amount the young that we observed could be attributed to several possible factors such as the economic challenges due to the financial crisis on the island, post-disaster trauma due to the recent hurricanes, and limited access to mental health services due to the economic and political upheavals [1, 2, 4].

The global shift towards an aging population has wide-ranging implications for society, including social, economic, political, and health considerations [29, 32]. It is crucial to study the primary sociodemographic and health factors affecting older adults’ health and perceived HRQoL, given the rise in the percentage of people over 65 and the focus on “good/healthy aging” [29]. Puerto Rico faces a growing number of elderly residents, a rise in chronic illnesses [22] as Puerto Rico becomes a destination hub for retirees, and young people leave the island for better economic opportunities. Aging often leads to a decline in functional abilities and increased healthcare needs, rendering older adults more vulnerable to chronic conditions [32]. Maintaining optimal levels of basic function and health is essential for promoting good aging, as a decrease in physical health negatively affects participants’ perception of the physical component of their HRQoL [28]. Additionally, with the increasing prevalence of diseases, disabilities, dementia, and dysfunctions in old age, extending lifespan alone may not be sufficient [33, 34]. These circumstances have profound implications for population health of Puerto Ricans, including increased strain on the healthcare system, higher healthcare expenses, and greater demand for healthcare services.

Low household income is another significant sociodemographic factor influencing the HRQoL of adults in Puerto Rico. Our study found that low income is associated with reduced HRQoL in all HRQOL-4 domains, which aligns with previous research [30]. Additionally, our study also suggested that regardless of education or employment status, low income increases the likelihood of reporting *poor HRQoL*, especially in the general health domain. Since the last time HRQoL was measured in PR in the last decade, the prevalence of low-income individuals rating HRQoL as *poor* has not improved [12]. The rising poverty levels in the context of Puerto Rico could result in increased rates of chronic diseases, reduced healthcare access and utilization, decreased mental well-being, and overall diminished HRQoL for those affected [22, 34]. The literature on health disparities shows that low-income individuals may have difficulty accessing health services due to competing demands like seeking employment, working multiple jobs, caring for family, and making tough financial choices between healthcare and basic necessities such as food or housing [30]. Due to various factors such as income and wealth inequality, natural disasters, and limited access to health and social welfare infrastructures [4, 34], it is reasonable to assume that those with lower incomes in Puerto Rico report lower HRQoL. Interventions such as access to healthcare services, chronic disease management initiatives, mental health services expansion, physical activity promotion and population health policy and research efforts must evaluate the association and impact of sociodemographic characteristics and chronic conditions on the HRQoL of adults living in Puerto Rico, considering the different contexts that affect an individual, interpersonal, and population level of the island.

### Limitations and strengths

The BRFSS survey is limited by its self-reported, cross-sectional nature, preventing inferring causal relationships between *poor HRQoL* and other respondent characteristics. Additionally, as a telephone survey, it excludes homeless and lower socioeconomic status individuals who may have more health impairments, as well as adults living in nursing homes or long-term care facilities who tend to have lower HRQoL [15]. This exclusion may introduce selection bias and underestimation of *poor HRQoL*. Also, self-reported data may be subject to response bias and under or misreporting health behaviors, conditions, and HRQoL.

Despite these limitations, our study has several strengths. To the best of our knowledge, this is the first study utilizing the BRFSS that assesses the association between the four HRQoL-4 domains and sociodemographic characteristics and chronic conditions among adults living in Puerto Rico. The BRFSS gives reliable and valid results as it is designed to provide broad-based population information and relate to the HRQoL-4 domains [22, 30]. Also, the use of a representative dataset makes it possible to generalize our findings of HRQoL perceptions to the Puerto Rican adult population. Finally, investigating the four HRQOL measures together is an additional strength of the current study.

## Conclusion

Through a comprehensive multivariable analysis, we have uncovered significant risk factors associated with *poor HRQoL* among adults living in Puerto Rico. These factors include chronic conditions, age, and income. Specifically, our findings suggest that individuals with an increasing number of chronic conditions were more likely to report *poor HRQoL* across all four domains. In addition, middle-aged and older adults, as well as those with lower socioeconomic status, were at increased risk for *poor HRQoL*. Furthermore, the prevalence of people rating their HRQoL as *poor* shows that it has not improved since it was last assessed in 2011 [12]. The challenges facing Puerto Rico, such as fiscal austerity, income inequality, a debt crisis, significant emigration, and a suboptimal healthcare system, only exacerbate these disparities. Investigating potential patterns in HRQoL measures is crucial to better plan for future healthcare needs, changing demographics, and environmental and economic landscapes.

Using multiple domains of HRQoL can help to assess the burden of poor health in.

a population, identify subgroups with unmet HRQoL needs, inform the development of targeted interventions, and monitor changes in a population’s HRQoL over time [35]. The use of these HRQoL domains in longitudinal and intervention studies is needed to increase our understanding of the causal relationships between sociodemographics, health risk factors, and HRQoL. Priority should be given to improving socioeconomic status, promoting healthy lifestyle behaviors, and addressing chronic conditions. Thus, governmental actions are necessary to lessen the burden on older adults, low-income adults, and adults with chronic conditions in Puerto Rico.

## Data Availability

The 2019 Behavioral Risk Factor Surveillance System (BRFSS) data files supporting the conclusions of this article are publicly available at the website https://www.cdc.gov/brfss/annual_data/annual_2019.html.

## Abbreviations

HRQoL: Health-related Quality of Life
HRQoL-4: Healthy Days Measures
BRFSS: Behavioral Risk Factor Surveillance System
US: United States
CDC: Centers for Disease Control and Prevention
POR: Prevalence Odds Ratio
CI: Confidence Intervals
Ref: Reference group
